# Leader’s Machiavellianism and employees’ counterproductive work behavior: testing a moderated mediation model

**DOI:** 10.3389/fpsyg.2023.1283509

**Published:** 2024-01-18

**Authors:** Han Cai, Le Wang, Xiu Jin

**Affiliations:** Department of Business Administration, Gachon University, Seongnam-si, Republic of Korea

**Keywords:** counterproductive work behavior, Leader’s Machiavellianism, moderated mediation model, organizational political behavior, perceived abusive supervision

## Abstract

Counterproductive work behavior wastes organizational resources and significantly damages organizational development. The importance of employees’ counterproductive work behaviors in organizations is becoming increasingly obvious. This is directly related to the sustainable development and survival of organizations. This study believes that employee’s behavior is closely related to leadership style. In particular, employees’ in small- and medium-sized enterprises are often manipulated and deceived by leaders, resulting in dissatisfaction and counterproductive work behavior. In order to address this behavior, this study collected survey data from 289 employees from Chinese SMEs to explore the relationship between perceived abusive supervision and organizational political behavior in Machiavellian leadership and counterproductive work behavior. The results suggest that Machiavellian positive influence counterproductive work behavior through a mediating role of perceived abusive supervision. Furthermore, leader organizational political behavior moderates the indirect effect of perceived abusive supervision such that the effect is stronger when leader organizational political behavior is high. This study aimed to identify the variables that increase employees counterproductive work behavior, propose recommendations for reducing employees’ counterproductive work behavior, expanded the scope of counterproductive work behavior research, and provided a theoretical basis for related studies.

## 1 Introduction

With the reform of the enterprise organizational structure and increasing employees’ autonomy and their negative behavior are becoming increasingly popular in the working environment with increasing degrees of freedom (Wang et al., 2022). Rapid change in the organizational environment increases employees’ job stress, which leads to counterproductive work behavior (Chen et al., 2022). With its characteristics of high generality and concealment, it is widespread in organizations and has become a problem faced by most enterprises today (Wang et al., 2022). Counterproductive work behavior refers to employees deliberately engaging in unethical, illegal, or other unwelcome behaviors (Sulea et al., 2013). Counterproductive work behavior not only affects the effectiveness of the organization but also creates an atmosphere of mistrust and negative emotions, which in turn affect work and employee well-being (Rehman and Shahnawaz, 2018). It also brings high financial costs to the organization and adversely affects employees (Kessler et al., 2010). Therefore, these highlights show the importance of counterproductive work behavior in an organization, which should reduce counterproductive work behavior to create a positive and trusting working atmosphere.

In addition, according to previous researches, organizational atmosphere, culture, leadership style, and other factors affect employees’ behavior. In organizations, leaders’ care and support for employees is an important manifestation of employee learning and development (Su et al., 2019), and individual and organizational performance depend on the role and influence of the leader, who influences the attitudes and behaviors of subordinates (Cai and Jin, 2023). This study predicts that leadership is a key factor in improving counterproductive work behavior. In particular, the Machiavellian leadership style is directly related to counterproductive work behavior. Machiavellianism refers to the personality trait of using others to achieve success; the core concept of this personality is the achievement of personal goals through manipulative and persuasive behavior (Chen, 2010). Machiavellian leaders are adept at using various unethical means to harm employees when personal interests are involved to achieving their goals (Özsoy, 2018). According to prior research, there is a positive correlation between employees’ performance appraisal justice, work engagement, and organizational identification in the organization (Lyu et al., 2023). In other words, when employees feel the leader’s fairness in the organization, they will improve the organization’s a sense of identity makes it easier to devote yourself to work. When employees are confronted with Machiavellian leadership, they fear that their interests will be violated; thus, out of their sense of self-protection, they will act negatively on their own initiative. According to the conservation of resources theory, individuals strive to hold, protect, and develop personal resources in the process of interacting with the environment (Liu et al., 2023). Additionally, individuals with high Machiavellian tendencies pay more attention to personal interests, exhibit opportunistic behavior to maximize benefits and engage in immoral behavior, thus negatively impacting the organization (Zhao et al., 2018). In such a situation, employees are vulnerable and can develop counterproductive work behaviors. Therefore, we believe that Machiavellian leadership increases employees’ counterproductive work behaviors.

This study aimed to test whether Machiavellian leadership enhances counterproductive work behavior and whether perceived abusive supervision mediate the relationship between leadership Machiavellianism and counterproductive work behavior. Abusive supervision is a subjective evaluation of subordinates’ superiors’ behavior (Hoobler and Brass, 2006). Abuse of supervision means that the quality of the relationship between employees and leaders is poor, and employees have no obligation to participate in behaviors that promote organizational goals or show a higher level of organizational commitment (Aryee et al., 2007). In addition, abusive supervision positively affects counterproductive work behavior (Eschleman et al., 2014). Specifically, when a person belongs to and is identified with a certain group, evaluations of group-related events or characteristics will trigger a person’s group emotions (Hou et al., 2021), thereby increasing the incidence of triggering behaviors. Therefore, in an organization, when leaders engage in immoral behaviors such as manipulation and deception toward employees for personal benefit, employees’ perceived leaders’ abusive supervision increases, thus leading to counterproductive work behavior. Therefore, Machiavellianism positively impacts counterproductive work behavior through perceived abusive supervision.

In addition, this study argues that changes in employees’ counterproductive behavior vary with the effect of the moderated mediation of the leader’s organizational political behavior. Leaders’ political behavior creates a negative organizational atmosphere, which has a negative impact on employees’ attitudes and behavior, and the degree of influence depends on employees’ perceptions (Huang and Du, 2022). When employees perceive the success that leaders have achieved by engaging in political behavior, they decrease their reputation and trust in the leader, which leads to less concern for the organization and negative behaviors (Ferris et al., 2000). This study believes that organizational political behavior strengthens perceived abusive supervision and provides reasons for their counterproductive work behavior. Therefore, it is necessary to explore the regulatory effect of political behavior and the interaction between perceived abusive supervision and leaders’ organizational political behavior, which will increase counterproductive work behavior.

Based on the above theories, the purpose of this study is summarized as follows: First, a compilation of previous research findings confirmed the relative lack of empirical research on Machiavellian leadership and counterproductive work behavior in China at present. Therefore, we elucidated the relationship between Machiavellian leadership and counterproductive work behaviors. It shows how Machiavellianism leads to counterproductive work behavior, which will help expand the field of research on counterproductive work behavior.

Second, most studies have explored the antecedents of counterproductive behavior (Li, 2022; Wang et al., 2022; Xia and Wang, 2022), and verified their mediating or moderating role in inducing counterproductive behavior (Nikkah-farkhani et al., 2017; Rehman and Shahnawaz, 2018; Qin et al., 2022) However, we have expanded the scope of counterproductive behavior research. In addition, we propose and verify a moderated mediation research model.

Third, most studies treat organizational political behavior as an independent or subordinate variable; however, this study identified and examined the moderating role of organizational political behavior. Specifically, by presenting the interaction between perceived abusive supervision and organizational political behavior, we identified how interaction effects change counterproductive work behavior, and thus moderate the mediating effect of perceived abusive supervision.

Fourth, in past research, studies of Machiavellianism have typically focused on populations such as children (Yang et al., 2017), student (Wu and Shen, 2011), and airport work employees (Xu and Luo, 2018), but relatively little attention has been paid to the workplace, with more limited research especially among employees in SMEs. The workplace serves as a social environment where leadership regularly interacts with employees and is able to monitor employee performance and behavior (Shoss et al., 2015). Therefore, this study focuses on SME employees to examine the impact of Machiavellianism on employee behavior and, in turn, the impact it has on the organization.

Finally, we lack research on the counterproductive work behavior in Chinese SME, and we elucidate the role of counterproductive work behavior in Chinese SMEs. This attempt will help expand the field of counterproductive work behavior research. Specifically, this study proposes a new research model for increasing counterproductive work behavior and reveals how Machiavellian leadership leads to counterproductive work behavior. It contributes to expanding the research field of Machiavellianism and counterproductive work behavior. Furthermore, it will reveal the extent of leaders’ organizational political behavior in Chinese SMEs and help us understand the role of organizational political behavior in Chinese SMEs through interaction with perceived abusive supervision.

## 2 Theoretical background and hypotheses development

### 2.1 Machiavellianism

The constitutive concept of Machiavellianism is derived from the 16th-century political theorist Machiavelli (Kim et al., 2011). Machiavellianism is a selfish, apathetic trait that allows individuals to deceive and manipulate others to maximize their own interests (Koo et al., 2016). The Machiavellian individual’s lack of trust and tendency to manipulate others clearly violates the basic principles of reciprocity, trust, and cooperation in interpersonal exchange, thus producing negative impacts in organizations (Yang et al., 2023). Moreover, Machiavellianism is defined as the tendency to manipulate others, focus on self-interest, avoid emotional expression, and deviate from typical moral norms to control others because of indifference and deception about others’ rights (Ricks and Fraedrich, 1999; Chung and Shin, 2021). Therefore, Machiavellianism has a major negative impact on work behavior and attitude (Kim et al., 2022). When leaders with strong Machiavellian tendencies cheat, employees’ morale decreases, generating many passive work attitudes, eventually causing talent to leave (Chung and Shin, 2021). Therefore, Machiavellianism is likely negatively impact organizational performance and interpersonal relationships (Park et al., 2022).

Machiavellian personality traits are opportunistic; Machiavellian leaders are likely to betray the organization or partners frequently for their own interests, which not only destroys efficiency within the organization but may also cause serious harm to the organization (Kim et al., 2011). Machiavellian leadership refers to the use of radical, manipulative, exploitative or cunning means to achieve personal and organizational goals (Fraedrich et al., 1989). Machiavellian leaders, to maximize their own interests, are likely to betray others and act selfishly; when they are betrayed, they show a strong desire to punish and retaliate against others (Koo et al., 2016). Machiavellianism is defined as a strategy of social behavior that requires influencing others to achieve personal interests, often against the interests of others (Hammali and Nastiezaie, 2022). Leaders with a high level of Machiavellianism will skillfully adjust their words and deeds according to the situation, thereby effectively covering up the dark side of their personality and achieving the purpose of manipulating others (Yang et al., 2023).

Machiavellianism has been found to positively impact unethical pro-organizational behavior (Kim et al., 2022) and employees’ turnover intention (Chung and Shin, 2021). Machiavellianism positively impacts counterproductive work behavior (Park et al., 2022) and organizational political perceptions (Lee et al., 2016). Moreover, Machiavellianism positively impacts abusive supervision (Kiazad et al., 2010).

Therefore, leaders with high Machiavellianism will deceive and manipulate employees for their own interests, and employees will lose their enthusiasm for work, leading to counterproductive work behavior.

### 2.2 Perceived abusive supervision

Innovative behavior serves as the key to organizational development and success. In organizations, employees with high internal motivation tend to focus on their tasks and tend to be immersed in service work, but when employees perceive abusive supervision, they are unwilling to put themselves available resources are spent on tasks that require creative effort and investment, thereby bringing negative results to the organization (Su et al., 2020; Akram et al., 2022). Abusive supervision refers to superiors engaging in hostile verbal and non-verbal behaviors other than physically harming their subordinates (Nam and Yoo, 2016). Abusive supervision refers to employees’ understanding of the extent to which leaders continue to exhibit hostile verbal and non-verbal behaviors (except physical contact; Tepper, 2000). When they feel abusive supervision by the leader, they will have negative emotions such as unease, complaints, and dissatisfaction, thus reducing their enthusiasm and work ethic (Zhao, 2022). Moreover, the higher the level of abusive supervision, the lower the trust in their leaders; therefore, when employees’ perceive abusive supervision, they increase negative awareness, resulting in negative attitudes and behaviors (Du and Jang, 2022). Abusive supervision refers to behavior in which leaders ignore subordinates with verbal and non-verbal actions, lose face in front of others, and deliberately ignore subordinates’ opinions (Choi et al., 2022). In an organization, employees’ perceptions of leaders’ continuous verbal or non-verbal intentional behavior significantly negatively impacts their psychology and behavior (Li et al., 2013).

Abusive supervision refers to the extent to which employees subjectively view a leader’s leadership and the persistent hostile words, deeds, and nonverbal behaviors (except for physical contact) toward them (Gatti et al., 2019). Abusive supervision includes the constant expression of nonphysical hostility (Tepper, 2007). Therefore, abusive supervision weakens the will and tension of employees, reduces their own organizational citizen behavior, and negatively impacts organizations (Zhao, 2022). Employees’ awareness of a leader’s non-personality behavior affects their understanding of the organization, thus reducing their job and life satisfaction, organizational input, and other behaviors, and increasing psychological stress (Tepper, 2000). Employees will feel psychological pain due to abusive supervision by leaders, which will lead to a decrease in creativity levels (Akram et al., 2022).

A study on abusive supervision found that it negatively impacts organizational citizenship behavior (Zhao, 2022) and leadership trust (Du and Jang, 2022). Abusive supervision was positively associated with psychological distress (Akram et al., 2022). Machiavellian Leadership positively impacts on perceived abusive supervision (Kiazad et al., 2010). Distributive and interactive justice negatively impact abusive supervision (Nam and Yoo, 2016). Moreover, low-level perceived LMX positively impacts perceived abusive supervision (Martinko et al., 2011).

Based on the above theory, this study holds that perceived abusive supervision refers to perceptions of the hostile behavior of leaders’ continuous nonphysical contact. Therefore, when employees perceive abusive supervision of the leader, they lose trust in the leader and their concern for the organization.

### 2.3 Organizational political behavior

Organizational politics is prevalent in all organizations, to varying degrees (Khattak et al., 2023). Organizational political behavior refers to behavior that influences others or groups to obtain and protect the interests of individuals and related groups under the domination of potential motivation (Ma et al., 2006). Organizational political behavior is behavior that achieves an end through means of influence that is not approved by the organization (Mayes and Allen, 1977). Leaders’ participation in organizational political behavior reduces employee satisfaction and interest in the organization, leading to employee silence (Liu and Jing, 2008). Moreover, when employees perceive the leader’s organizational political behavior, it reduces their self-confidence and trust in the organization, increases the turnover rate, and negatively impacts the organization (Sun, 2013). Organizational political behavior is a kind of behavior that is not explicated or is difficult to clearly define in the organizational system, which defines the behavior generally as the use power or other resources to influence others or groups in pursuit of their own interests (Qiu, 2015). Organizational political behavior serves as a hindrance stressor among employees because it hinders employees’ ability to achieve goals and obtain resources in the workplace (Khattak et al., 2023). Therefore, in an organization, leaders use non-business authority or resources to intervene or influence on employees to achieve their goals. In the long run, it reduces employees’ trust in leaders and negatively impacts organizations (Chen et al., 2016).

Organizational political behavior refers to the behavior in which individuals or organizations obtain, develop, and use power and other resources to achieve the desired results (Sun, 2013). Organizational political behavior is behavior that, although not part of the organizational role, influences or attempts to influence activities within the organization (Farrell and Petersen, 1982). Therefore, under the conditions of resource scarcity and information asymmetry, some organizational political behaviors negatively impact (sense of injustice) in different ways, resulting in more organizational political behaviors, perpetuating a vicious circle (Lu and Gu, 2009). Moreover, leaders who are good at political behavior in the organization will lead the company to evaluate employees only according to their feelings; in the long run, truly capable employees can easily lose trust in their leaders and confidence in the development of the company, eventually leading to brain drain (Qiu, 2015). Therefore, in organizations, high levels of political behavior will pose a threat to employees’ resources and produce negative results for employees’ attitudes and performance (Khattak et al., 2023).

A study of organizational political behavior found that it positively impacts organizational silence (Liu and Jing, 2008). Machiavellianism positively impacts organizational political behavior (Chen et al., 2016). Organizational input negatively impacts organizational political behavior (Yılmaz et al., 2014). Moreover, general political behavior negatively impacts performance indicators (Lee et al., 2016). Relationship-oriented organizational culture negatively impacts political behavior within an organization (Cheong, 2015).

According to this theory, this study holds that organizational political behavior refers to the behavior of individuals or groups using various means of profit. Therefore, the higher leaders’ level of political behavior, the more uneven the distribution of resources in the organization, and the more likely employees are to perceive the organization as unjust and finally complain to the company.

### 2.4 Counterproductive work behavior

Counterproductive work behavior is the harmful spontaneous behavior of an individual at work that intentionally harms the legitimate interests of an organization, its internal employees, and external stakeholders (Lv, 2015). Counterproductive work behavior refers to all types of biased organizational behavior that negatively impacts an organization (Park et al., 2015). At workplace, counterproductive work behavior leads to the loss and waste of organizational resources, resulting in a decline in work quality (Nikkah-farkhani et al., 2017). In the long run, it negatively impacts colleagues or the organization and prevents the organization from performing its duties normally, resulting in serious consequences for the organization (Lv, 2015). Counterproductive work behavior refers to intentional behaviors by employees that harm the organization or its members (Yan et al., 2022). If employees engage in counterproductive work behavior (such as slack work) because of negative emotions, this may harm the organization’s operations and management (Wang et al., 2022). Counterproductive work behavior can be regarded as behavior that violates norms and rules, reduces the development and security of the organization and members, and intentionally causes harm (Minjeong and Hoon, 2023). Counterproductive work behavior is a potentially destructive behavior that is harmful to both individuals and organizations, and will have a negative impact on organizational performance (Qu and Lee, 2021).

Counterproductive work behavior refers to employees intentionally harming their organization or its employees (Penney and Spector, 2002). Counterproductive work behaviors are voluntary and potentially destructive behaviors of employees that reduce organizational productivity, climate, and outcomes (Lv, 2015). Counterproductive work behavior refers to behaviors in which employees intentionally damage the welfare of an organization (Putra and Putra, 2022). Therefore, counterproductive work behavior reflects the negative attitudes toward work and negatively affects both organizations and individuals (Kim and Yoon, 2017).

A study on anti-production behavior found that person–organization fit negatively impacts counterproductive work behavior (Nikkah-farkhani et al., 2017). Incivility positively affects counterproductive work behavior. Employees’ job burnout positively impacts counterproductive work behavior (Park et al., 2015). Organizational silence positively impacts counterproductive work behavior (Shin, 2020). As an employee’s counterproductive work behavior that violates the organization’s rules, regulations or values, it not only causes damage to the organization, but also damages the trust relationship with their superiors (Xia and Wang, 2022). Moreover, counterproductive work behaviors positively impact work complexity (Kim and Yoon, 2017).

According to this theory, this study holds that counterproductive work behavior refers to spontaneous behavior in which deliberately damage the interests of the organization because of its negative influence. Therefore, employees’ anti-production behavior destroys an organization’s normal order and working atmosphere, reducing its productivity.

### 2.5 Machiavellianism and perceived abusive supervision

In an organization, when Machiavellian leaders distrust employees, manipulate and exploit them for their own interests, or even deceive them, employees feel abusive supervision from their leaders (Khan et al., 2023). In addition, leaders with Machiavellian behavioral characteristics are more likely to exhibit aggressive behavior toward employees and do not help them release negative emotions, leading to perceptions of leaders’ abusive supervision (Zhang and Bednall, 2015). When leaders have Machiavellian personality traits, they form widespread aggressive behaviors in the organization, leading to an increase in employees’ perceived abusive management by leaders (Wu et al., 2022). Through manipulation and control, Machiavellian leaders let employees participate in work-related activities without willingness or interest, reduce their enthusiasm for work commitment, and increase their long-term perception of leaders’ abusive supervision (Teng et al., 2021). Individuals with high Machiavellian traits may use aggressive, profitable, and deviant behaviors to achieve personal and organizational goals, paying less attention to the welfare of others, resulting in employees experiencing abusive supervision by their leaders (Hammali and Nastiezaie, 2022). Leaders with high levels of Machiavellianism ignore ethics to achieve personal goals, appear emotionally cold and distrustful of others, resulting in a lack of close relationships with employees and, in this case, resulting in employees feeling to abusive supervision by leaders (Hammali and Nastiezaie, 2022). Moreover, Machiavellian leaders abuse employees because their competitive worldview creates a negative working atmosphere for the organization, thus increasing perceived abusive supervision (Khan et al., 2023). Based on these theories, this study proposes the following hypotheses:

*Hypothesis 1:* Leader’s Machiavellianism positively influences perceived abusive supervision.

### 2.6 Machiavellianism and counterproductive work behavior

At work, when employees feel that their leaders have Machiavellian tendencies, they feel uneasy because of the uncertainty of the environment, may be strongly aware of their interests being thwarted, and show counterproductive work behavior to preserve their interests and resources (Park et al., 2022). Based on the employee-organization relationship theory, leaders’ Machiavellianism can affect employees’ counterproductive work behavior (Minjeong and Hoon, 2023). Moreover, Machiavellian leaders are more likely to participate in operational behavior when faced with achieving goals; are very impulsive in interpersonal communication; and are more likely to lie, deceive, mislead, and use the loyalty of employees to their own benefit, resulting in reduced trust in the organization and counterproductive work behaviors (Amir and Malik, 2016; Li et al., 2020). In an organization, a leader with Machiavellianism will behave like manipulating and exploiting employees. In the long run, employees will feel this behavior of the leader, which will lead to employees becoming dissatisfied with the organization through depression, anger, etc. Negative emotions exhibit counterproductive work behavior (Minjeong and Hoon, 2023). In addition, Machiavellian leaders act impulsively and irresponsibly during interpersonal interactions, creating a negative working atmosphere for the organization over a long period and leading to counterproductive work behavior (Baloch et al., 2017). Machiavellian leadership is insufficient in the ability to establish healthy interpersonal relationships and positive behaviors, and can easily provide a psychosocially stressful environment in the workplace, which has never led to the occurrence of unwanted sexual behavior among employees (Hammali and Nastiezaie, 2022). Based on these theories, this study proposes the following hypotheses:

*Hypothesis 2:* Leader’s Machiavellianism positively influences counterproductive work behavior.

### 2.7 Perceived abusive supervision and counterproductive work behavior

Loud reprimands, insults, and public ridicule and belittling of employees are typical abusive supervision behaviors. When employees feel the abusive supervision of their leaders, they will generate psychological pressure and release a certain amount of pressure through counterproductive behaviors (Lu et al., 2020). Leaders’ abusive supervision leads to a decrease in perceived organizational support, thus reducing their motivation to help the organization achieve its goals; therefore, when employees perceive abusive supervision, they associate it with the organization, believing that the organization mistreats them, and retaliate accordingly, leading to counterproductive work behavior (Shoss et al., 2013). As a stressful situation, abusive supervision will have a negative impact on employees’ emotions. In this situation, employees will use their own resources to control their emotions, thereby increasing the occurrence of counterproductive behaviors of employees (Lu et al., 2020). Employees perceive abusive supervision as a negative manifestation of the organization; therefore, when employees perceive abusive supervision in the organization, they exhibit negative behaviors and refuse to contribute positively, thereby producing high levels of counterproductive work behavior (Shoss et al., 2013). In addition, when employees perceive abusive supervision by their leaders, they become less dependent on the organization, generating counterproductive work behaviors, such as quitting, production deviations, sabotage, and theft (Wei and Si, 2013). In an organization, when employees feel the abusive supervision of their leaders, it will cause employees to feel unfit at work and have a psychological imbalance, thereby resisting the leaders’ decisions and producing counterproductive behaviors (Lu et al., 2020). Abusive leaders reduce autonomous motivation and increase counterproductive work behavior by reducing job satisfaction and innovative behavior (Ronen and Donia, 2020). Based on these findings, this study proposes the following hypotheses:

*Hypothesis 3:* Perceived abusive supervision positively influences counterproductive work behavior.

### 2.8 The mediating effect of perceived abusive supervision

Leaders who suppress or inhibit the performance of others and stimulate others to engage in negative behaviors are more likely to engage in abusive supervision, according to previous research findings (Wisse and Sleebos, 2016). Specifically, Machiavellianism, as a humanly negative view and behavior, centers on ignoring ethical norms and extracting great benefits from others through unethical use, exploitation, or deception (Deng et al., 2022). In addition, employees’ own outstanding achievements and performances will also attract the attention of Machiavellian leaders and even be seen as a threat, and the leaders will manage them through suppression, ostracism, and other abusive ways in order to keep their own interests intact (Khan et al., 2023). In the Machiavellian leadership style, the leader has little confidence in his employees, and the relationship between them is also based on intimidation and fear, in this case, the leader will force employees to complete specific tasks in a short period of time or to Employees exercise strict control, which leads to employees feeling the abusive management style of their leaders, and in this work environment, employees’ self-esteem will be reduced, work pressure will be increased, and the occurrence of counterproductive behaviors will increase (Hammali and Nastiezaie, 2022). Machiavellian leaders assert their position and control their subordinates’ performance by threatening and intimidating employees. In the long run, employees perceive these behaviors as abusive supervision and actively increase their level of counterproductive work behavior to protect their resources (Kiazad et al., 2010). Machiavellian leadership increases disagreements and conflicts with employees and contributes to employees’ perceived abusive supervision, leading to increased frustration, anger, aggression, and counterproductive work behavior (Brees et al., 2014). When employees feel mistreated in different ways by leaders in different positions, it will damage the employee’s image and ability, thereby weakening the employee’s self-efficacy and increasing the possibility of counterproductive behavior (Hammali and Nastiezaie, 2022). In addition, when leaders intentionally engage in abusive behaviors, they create discomfort and elevate employees’ negative emotions, leading to perceived abusive supervision, prompting strong resistance behaviors, and ultimately triggering the onset of counterproductive work behaviors (Schyns et al., 2018). Machiavellian leaders may use unethical behaviors, such as verbal aggression and disregard for extended periods, which can contribute to feelings of anxiety, fear, and tension among employees, resulting in perceived abusive supervision; when employees’ emotions lead to increased stress, they are more likely to engage in counterproductive work behaviors (Liu et al., 2018). Machiavellian leaders are able and even encouraged to use all available means to achieve selfish goals and will be self-centered, thereby establishing an unethical psychological work environment in the organization that leads to abusive management of employees’ perceived Increase, in this case, employees will vent their frustration by rejecting the leader’s decision-making, leading to an increase in counterproductive behavior (De Hoogh et al., 2021; Low et al., 2021). Based on these findings, this study proposes the following hypotheses:

*Hypothesis 4:* Perceived abusive supervision mediates the relationship between leader’s Machiavellianism and counterproductive work behavior.

### 2.9 The moderated mediation effect of organizational political behavior

This study emphasizes the moderating role of leaders’ organizational political behavior and argues that this variable strengthens the relationship between perceived abusive supervision and counterproductive work behavior. Thus, counterproductive work behavior in Chinese SMEs is determined by the interaction between their perceived abusive supervision abuse and leaders’ organizational political behavior. The essence of organizational political behavior is resources, power, and conflict (Chen et al., 2016). When leaders successfully use political behavior, they create a negative working atmosphere (Qiu, 2015). Moreover, organizational leaders play a critical role in the formation and transmission of organizational culture; therefore, leaders can easily form and spread an egoistic culture through organizational political behavior, which encourages employees to believe that personal interests are higher than organizational interests and reduces their concern for the organization (Liu and Jing, 2008). According to the conservation of resources theory, when leaders engage in political behaviors, such as cunning and manipulation, employees perceive leaders as acting contrary to what should happen in a rational and effective organization, leading to counterproductive work behaviors to achieve organizational goals (Dipboye, 2018). When leaders frequently engage in organizational political behavior, employees feel that all their efforts and inputs are useless and that their workplace circumstances are always determined by political behavior rather than values; they begin to feel stressed, leading to feelings of burnout, further leading to counterproductive work behaviors among employees (Makhdoom et al., 2017). Especially in Chinese SMEs, leaders’ organizational political behavior can negatively impact the organization, increasing employees’ counterproductive work behavior. Therefore, when leaders engage in political behavior in an organization, employees believe that they are not treated equally by the organization in terms of resource allocation. As a result, they become frustrated and engage in counterproductive work behavior (Ali et al., 2022). According to these theories, when employees perceive that their leaders use political behavior for their own benefit, they become frustrated with their leaders’ behavior and lose trust in them for extended periods, leading to increased counterproductive work behavior.

In addition, When the demand for resources in an organization exceeds the supply, the problem of rational resource allocation may become critical. As a result, leaders who tend to be authoritarian or have high power needs may control and manipulate others through political means to obtain more resources for their own interests, in this case, employees can easily feel the unfairness and selfishness of their leaders, leading to dissatisfaction and resentment toward their leaders’ implementation of oppressive management (Qiu, 2015). Therefore, leaders’ organizational political behaviors are expected to increase perceived abusive supervision among employees in Chinese SMEs. Leaders with organizational political behavior may treat employees abusively because they believe that by doing so, they can motivate employees to benefit the organization, in which case employees not only perceive leaders and organizations as political but also perceive leaders’ supervision as abusive (Liu and Liu, 2018). Increased political behavior within an organization can increase perceived abusive supervision by leaders, which increases their anxiety and insecurity (Kacmar et al., 2013). Additionally, leaders’ abusive supervision can create a sense of organizational unfairness, which, in turn, can influence employees’ attitudes and behaviors. Thus, employees who perceive abusive supervision for extended periods lose trust in the organization, have unproductive work attitudes, and are negligent toward their work, prompting counterproductive work behaviors (Ali et al., 2022). Leaders with a high level of political behavior use various means of benefiting themselves; such a political atmosphere causes employees to perceive leaders’ supervision as abusive, leading to high work pressure and counterproductive work behavior among employees. Therefore, increased organizational political behavior by leaders increases perceived abusive supervision by employees, increasing stress and counterproductive work behavior. Leaders who tend to emphasize their own interests are more adept at using destructive tactics, and Machiavellian leaders use employees to achieve their own goals through, which erodes employees’ trust in leaders and increases counterproductive work behavior (Elbers et al., 2023). This study highlights the mediating effect of organizational political behavior on the relationship between perceived abusive supervision and Machiavellianism and counterproductive work behavior. Leaders’ use of deception and political tactics for their benefit increases perceived abusive supervision and counterproductive work behavior. Overall, the higher the organizational political behavior of Chinese SME leaders, the more significant the effect of Machiavellianism on perceived abusive supervision and counterproductive work behavior.

Therefore, this study used perceived abusive supervision as a mediating variable between Machiavellianism and counterproductive work behavior. Organizational political behavior was used as a moderating variable between Machiavellianism, perceived abusive supervision, and counterproductive work behavior. This study emphasizes that organizational political behavior moderates the mediating effect of abusive supervision among Chinese SME employees. Thus, this study proposes the following hypotheses:

*Hypothesis 5:* Organizational political behavior positively moderates the relationship between perceived abusive supervision and counterproductive work behavior.

*Hypothesis 6:* The mediating influence of perceived abusive supervision on the relationship between leader’s Machiavellianism and counterproductive work behavior is moderated by organizational political behavior.

## 3 Research methodology

### 3.1 Sample characteristics

The samples of this study come from Chinese SMEs enterprises, and the employees of Chinese SMEs enterprises are the research subjects. In the process of data collection, we used an online questionnaire. A total of 289 responses were collected and used for the empirical analysis. With the consent of the individual, we ensure that all survey respondents fill out the survey voluntarily. Regarding the demographic characteristics of respondents, 177 (61.2%) were male, and 112 (38.8%) were female. Regarding age, 3 (1.0%) participants were under 20, 119 (41.2%) were 20 to 29, 95 (32.9%) were 30 to 39, 44 (15.2%) were 40 to 49, and 28 (9.7%) were 50 or older. Regarding education, 49 (17.0%) had completed technical secondary school or high school, 74 (25.6%) were junior college graduates, 122 (42.2%) were college graduates, 27 (9.3%) had master’s degrees, 8 (2.8%) were doctors, and 9 (3.1%) had other education. In terms of employment relationships, full-time jobs were the most numerous at 241 (83.4%), and informal positions were 48 (216.6%).

Regarding Service Years, 38 (13.1%) had worked for a year or less, 49 (17.0%) had worked for 1 to 3 years, 45 (15.6%) had worked for 3 to 5 years, 22 (7.6%) had worked for 5 to 7 years, and 135 (46.7%) had worked for seven or more years.

Regarding the time spent working with the current immediate leader, 69 (23.9%) had worked for a year or under, 60 (20.8%) had worked for 1 to 2 years, 58 (20.1%) had worked for 2 to 3 years, 28 (9.7%) had worked for 3 to 4 years, 11 (3.8%) worked with the current immediate leader for 4 to 5 years, and 63 (21.8%) had worked with the current immediate leader for five or more years.

Regarding enterprise type, 23 (8.0%) people worked in education, 33 (11.4%) in finance, 120 (41.5%) in coal mining, 16 (5.5%) in catering services, 5 (1.7%) in the medical industry, and 92 (31.8%) in other occupations.

### 3.2 Measurements and main variable

Machiavellianism is a personality trait characterized by distrust of others and the use of immoral means to control others for one’s own status and benefit (Dahling et al., 2009). This study used a tool mentioned by Dahling et al. (2009), which consists of 16 items, to measure the Machiavellianism of Chinese SME leaders. However, two items have been deleted for this study because the value was lower than 0.5. Sample items included “I like to give orders in interpersonal situations,” and “People are motivated only by personal gain.” Two items were removed because of low factor numbers.

Perceived abusive supervision refers to employees’ perception of leaders’ persistent hostile verbal or non-verbal negative behavior. This study used the tool used by Pradhan et al. (2019) to measure Chinese SME perceived abusive supervision by leaders. The measurement tool consists of five items, including “My supervisor ridicules me,” and “My supervisor makes negative comments about me to others.” To be more suitable for this study, the question item was changed to elicit responses on perceptions of abusive supervision.

Organizational political behavior refers to the behavior of an organization to promote its own interests, usually at the expense of the welfare of others or the organization (Kapoutsis, 2016). This study used the tool mentioned in Treadway et al. (2005) to measure Chinese SME leaders’ organizational political behavior. The measurement tool consists of six items, including “I use my interpersonal skills to influence people at work” and “I work behind the scenes to see that my work group is taken care of.”

**Figure 1:**
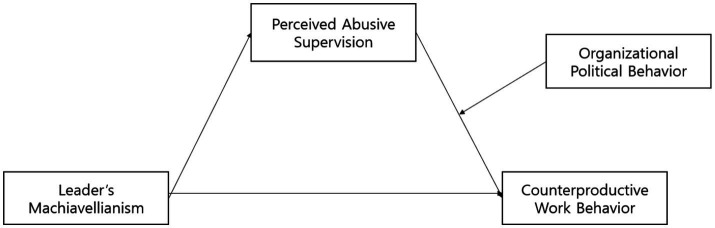
Research model.

Counterproductive work behavior refers to any behavior that employees deliberately carry out that potentially harms the legitimate interests of an organization or its members (Jiang et al., 2022). This study used the tool mentioned in Dalal et al. (2009). The measurement tool consisted of 12 items. Sample items included “I tried to harm my supervisor/a coworker” and “I did not work to the best of my ability.” All items were measured on a 7-point Likert scale (ranging from 1 = strongly disagree to 7 = strongly agree; Figure 1).

## 4 Data analysis

### 4.1 Confirmatory factor analysis and reliability analysis

The practicability of the different data models In this study was verified using confirmatory factor analysis (Presson et al., 1997). The results of the confirmatory factor analysis were as follows: The absolute fit indices were *X^2^* (*p*) =1749.29(0.000), *X^2^*/*df* = 3.011, and RMSEA = 0.084. The RMSEA is indeed a “badness of fit” index, with values very close to 0 indicating an almost perfect fit and greater RMSEA indicating a worse fit. For the RMSEA, values less than 0.05 reflect a small approximation error, values between 0.05 and 0.08 reflect an acceptable approximation error, and values greater than 0.10 constitute a poor model fit (Browne and Cudeck, 1992). Second, the incremental fit indices were IFI = 0.904 and CFI = 0.903. Third, the parsimonious-adjusted indices were PNFI = 0.753 and PGFI = 0.611.

This study analyzed the average variance extracted (AVE) and composite reliability (CR) values. Regarding AVE, Machiavellianism was 0.508, perceptions of abusive supervision was 0.623, organizational political behavior was 0.604, and counterproductive work behavior was 0.575; all values were greater than 0.5.

Regarding CR, Machiavellianism was 0.854, perceived abusive supervision was 0.829, organizational political behavior was 0.888, and counterproductive work behavior was 0.888, all greater than 0.7. The measurement has significant validity if the AVE of the variables is higher than 0.5 and the CR is higher than 0.7.

Reliability analysis measures the internal consistency of scale items (Qureshi et al., 2023). Therefore, this study also analyzed Cronbach’s alphas. For Cronbach’s alphas, Machiavellianism = 0.953, perceptions of abusive supervision = 0.922, organizational political behavior = 0.961, and counterproductive work behavior = 0.958; reliability analysis has significant validity if the Cronbach’s of the variables is higher than 0.7. Table 1 presents these results.

**Table 1:** The result of confirmatory factor analysis and reliability analysis.

Variables		Estimate	SE	C.R	*p*	Standardized regression weights	AVE	C. R	Cronbach’s alpha
Machiavellianism (A)	A1	1				0.591	0.508	0.854	0.953
	A2	1.154	0.050	23.264	***	0.713			
	A3	0.801	0.057	14.126	***	0.502			
	A4	0.978	0.064	15.324	***	0.608			
	A5	0.927	0.070	13.243	***	0.573			
	A6	1.217	0.054	22.460	***	0.806			
	A7	1.019	0.067	15.150	***	0.635			
	A8	1.229	0.050	24.380	***	0.839			
	A9	1.118	0.053	21.197	***	0.783			
	A10	1.203	0.048	24.835	***	0.847			
	A11	1.233	0.052	23.822	***	0.829			
	A12	0.907	0.051	17.617	***	0.701			
	A13	0.866	0.067	13.014	***	0.563			
	A14	1.229	0,049	25.136	***	0.851			
Perceived abusive supervision (B)	B1	1				0.824	0.623	0.829	0.922
	B2	0.885	0.058	15.222	***	0.625			
	B3	1.072	0.039	27.767	***	0.877			
	B4	1.047	0.042	25.207	***	0.841			
	B5	0.948	0.046	20.519	***	0.756			
Organizational political behavior (C)	C1	1				0.838	0.704	0.888	0.961
	C2	1.084	0.048	22.411	***	0.796			
	C3	1.182	0.058	20.211	***	0.796			
	C4	1.146	0.040	28.431	***	0.881			
	C5	1.133	0.040	28.647	***	0.883			
	C6	1.106	0.044	25.347	***	0.836			
Counterproductive work behavior (D)	D1	1				0.741	0.575	0.888	0.958
	D2	0.910	0.041	22.375	***	0.737			
	D3	1.030	0.055	18.768	***	0.728			
	D4	0.928	0.059	15.614	***	0.653			
	D5	0.963	0.064	15.139	***	0.666			
	D6	1.097	0.052	20.933	***	0.811			
	D7	1.090	0.059	18.343	***	0.761			
	D8	1.189	0.057	20.927	***	0.822			
	D9	1.207	0.055	22.067	***	0.838			
	D10	1.118	0.068	16.435	***	0.709			
	D11	1.206	0.055	21.828	***	0.834			
	D12	1.144	0.061	18.792	***	0.772			
Model fit index	*X^2^* (*p*) = 1749.290(0.000), *X^2^*/*df* = 3.011, RMSEA = 0.084, IFI = 0.904, CFI = 0.903, PGFI = 0.611, PNFI = 0.753								

***p < 0.001, **p < 0.01, *p < 0.05.

### 4.2 Descriptive statistics and correlation analysis

Table 2 shows the descriptive statistics and correlation analysis. Descriptive statistical measures included mean and standard deviation (SD). The means for Machiavellianism, perceived abusive supervision, organizational political behavior, and counterproductive work behavior were 3.662, 3.027, 3.152, and 2.786, respectively. In addition, the SDs of Machiavellianism, perceived abusive supervision, organizational political behavior, and counterproductive work behavior were 1.332, 1.286, 1.462, and 1.285, respectively.

**Table 2:** The results of descriptive statistics and correlation analysis.

	Mean	Standard deviation	Machiavellianism	Perceived abusive supervision	Organizational political behavior	Counterproductive work behavior
Machiavellianism	3.662	1.332	–			
Perceived abusive supervision	3.027	1.286	0.772***	–		
Organizational political behavior	3.152	1.462	0.868***	0.836***	–	
Counterproductive work behavior	2.786	1.285	0.652***	0.706***	0.694***	–

***p < 0.001, **p < 0.01, *p < 0.05.

This study conducted a correlational analysis to verify the correlation among the variables, the results of which are summarized as follows: Machiavellianism was positively associated with perceived abusive supervision (*r* = 0.772, *p* < 0.001), organizational political behavior (*r* = 0.868, *p* < 0.001), and counterproductive work behavior (*r* = 0.652, *p* < 0.001). Perceived abusive supervision was positively associated with organizational political behavior (*r* = 0.836, *p* < 0.001) and counterproductive work behavior (*r* = 0.706, *p* < 0.001). Moreover, organizational political behavior was positively associated with counterproductive work behavior (*r* = 0.694, *p* < 0.001).

### 4.3 Path analysis

SPSS Process Model 4 was used to analyze the mediation effect of perceived abusive supervision. The results show that Machiavellianism positively impacts perceived abusive supervision (estimate = 0.745, *p* < 0.001) and counterproductive work behavior (estimate = 0.255, *p* < 0.001). In addition, the results show that perceived abusive supervision significantly impacts counterproductive work behavior (estimate =0.500, *p* < 0.001). Therefore, Hypotheses 1, 2, and 3 were supported.

Hypothesis 4 proposed that perceived abusive supervision mediates the relationship between Machiavellianism and counterproductive work behavior. The indirect effect was 0.373. The bootstrapped confidence intervals were Boot LLCI = 0.262 and Boot ULCI = 0.478, as 0 was not included between Boot LLCI and Boot ULCI. These results indicate that the mediating effect of perceived abusive supervision is significant. This finding suggests that Machiavellianism increases counterproductive work behavior through perceived abusive supervision. Thus, Hypothesis 4 is supported. Table 3 presents the results of the path analysis.

**Table 3:** The results of process model 4.

Path			Estimate	S.E.	*t*	*p*	LLCI	ULCI
Machiavellianism	→	Perceived Abusive Supervision	0.745	0.036	20.5986	0.000	0.6741	0.8165
Machiavellianism	→	Counterproductive Work Behavior	0.255	0.061	4.185	0.000	0.1341	0.3773
Perceived Abusive Supervision	→	Counterproductive Work Behavior	0.500	0.064	7.823	0.000	0.3748	0.6269
**Indirect effect(s) of X on Y**								
Indirect Effect			Effect	Boot SE	Boot LLCI		Boot ULCI	
Machiavellianism→Perceived Abusive Supervision→Counterproductive Work Behavior			0.373	0.055	0.262		0.478	

### 4.4 Moderating effect of organizational political behavior

**Figure 2:**
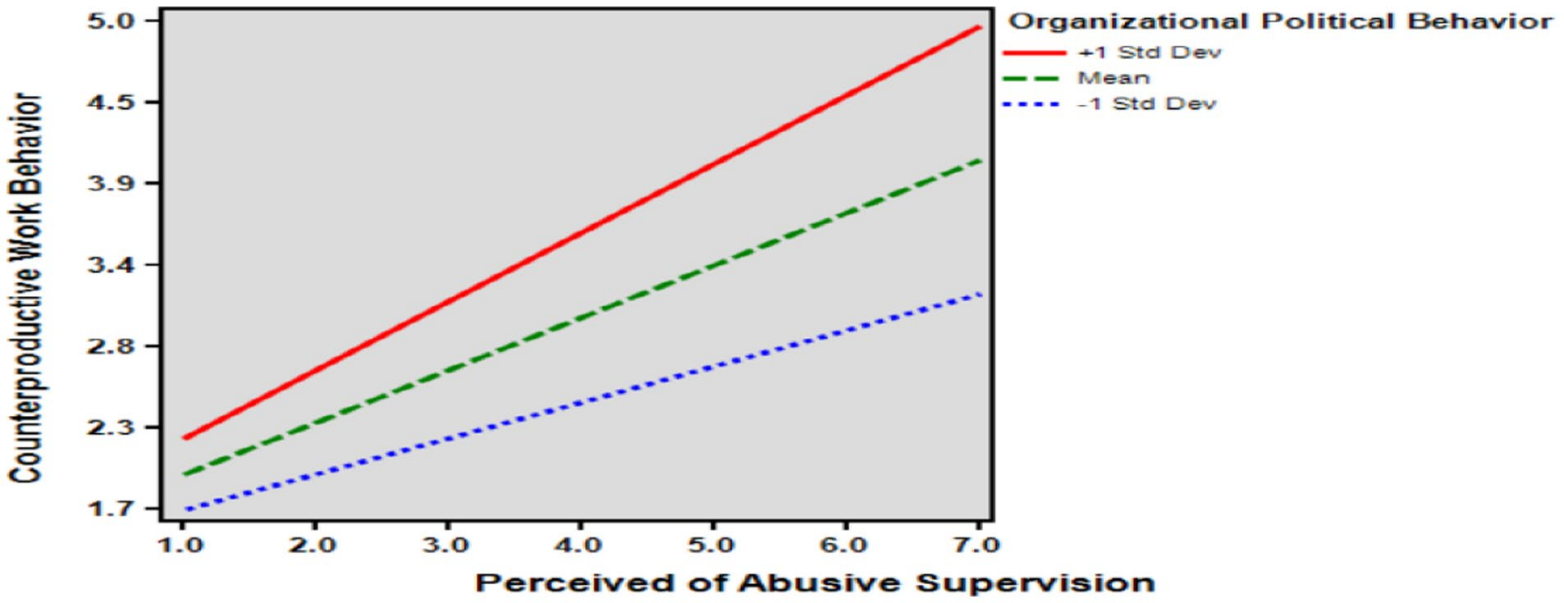
The moderating effect of organizational political behavior.

**Figure 3:**
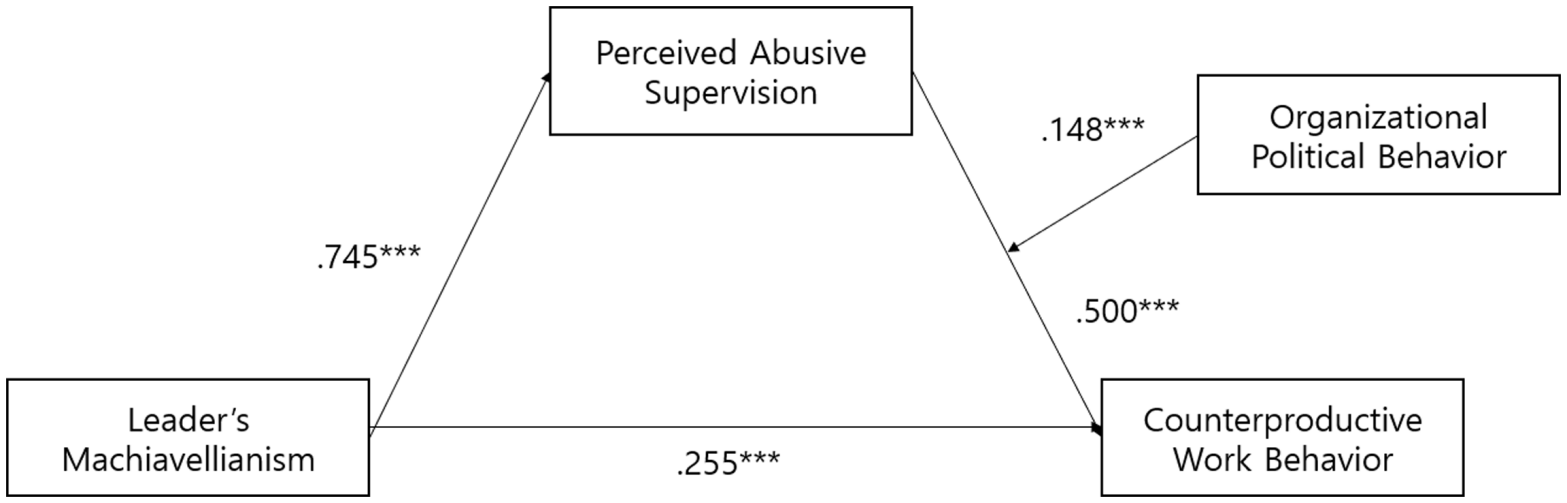
Fig with coefficients.

Hypothesis 5 established that organizational political behavior moderates the effect of perceptions of abusive supervision on counterproductive work behavior. The results showed that organizational political behavior significantly moderated the effect of perceived abusive supervision on counterproductive work behavior (*β* = 0.148, *p* < 0.001). Thus, the higher the organizational political behavior, the greater the impact of perceived abusive supervision on counterproductive work behavior. Therefore, Hypothesis 5 is supported. Therefore, the results show that the interaction between organizational political behavior and perceived abusive supervision increases counterproductive work behavior (Table 4; Figures 2, 3).

**Table 4:** The result of moderation.

Dependent Variable: counterproductive work behavior							
	Model 1		Model 2		Model 3		
	*β*	*t*	*β*	*t*	*β*	*t*	VIF
Perceived abusive supervision (A)	0.706***	16.880	0.417***	5.674	0.355***	4.792	3.507
Organizational political behavior (B)			0.346***	4.714	0.361***	5.010	3.324
Interaction					0.148***	3.529	1.126
R^2^(Adjusted R^2^)	0.498(0.496)		0.534(0.531)		0.554(0.549)		
△R^2^(△Adjusted R^2^)	–		0.036(0.035)		0.020(0.018)		
F	284.948***		164.116***		117.943***		

***p < 0.001, **p < 0.01, *p < 0.05.

### 4.5 Moderated mediation effect of organizational political behavior

Table 5 shows the moderated mediation effect on organizational political behavior. Hypothesis 6 established that organizational political behavior moderates the mediating influence of perceived abusive supervision on the relationship between Machiavellianism and counterproductive work behavior. The moderated mediation model was examined using SPSS PROCESS Macro 3.4.1 Model 14 and tested using 95% confidence intervals and 5,000 bootstrapping re-samples.

**Table 5:** The moderated mediation effect of organizational political behavior.

Dependent variable: counterproductive work behavior					
Moderator	Level	Conditional indirect effect	Boot SE	Boot LLCI	Boot ULCI
Organizational Political Behavior	−1 SD (−1.4626)	0.1559	0.0676	0.0229	0.2853
	M	0.2438	0.0591	0.1264	0.3562
	+1 SD (1.4626)	0.3317	0.0590	0.2117	0.4435
**Index of moderated mediation**					
	Index		Boot SE	Boot LLCI	Boot ULCI
	0.0601		0.0158	0.0252	0.0881

The conditional indirect effect of Machiavellianism on counterproductive work behavior was evaluated by analyzing the index of the moderated relationship at three different moderator levels: −1 SD, mean (M), and + 1 SD. Because 0 was not included between Boot LLCI and Boot ULCI at the level of −1 SD, mean level (M), and mean + 1 SD confidence intervals, statistical significance was confirmed.

Furthermore, the index of moderated mediation values was 0.0601, Boot SE =0.0158; Boot LLCI =0.0252, and Boot ULCI =0.0881. Because zero was not included between Boot LLCI and Boot ULCI, the bootstrapped confidence interval was significant. Therefore, Hypothesis 7 was supported.

## 5 Discussion

Many previous studies (e.g., Griep et al., 2022; Lee et al., 2022; Cao et al., 2023) has confirmed the serious harm that counterproductive work behavior brings to organizations and believes that reducing counterproductive work behavior in organizations it is an important task for organizations to achieve sustainable development. Although well established, little is known about the factor conditions underlying counterproductive work behavior. This study believes that leadership, as the organizer and leader in an organization, is an important factor in guiding employee behavior. Therefore, we took employees of Chinese SMEs as the research object and explored the relationship between Machiavellianism and counterproductive work behavior in more detail. The research of this study shows that leaders with high levels of Machiavellianism like to control others and will ask employees to do things for themselves in the face of interests. Employees working in this atmosphere will feel abusive supervision management by their leaders. Subsequently, employees’ perceived abusive supervision provides the necessary impetus for counterproductive work behavior and facilitates its occurrence. In other words, when employees’ perceived level of abusive supervision increases, counterproductive work behavior will also increase. Furthermore, the positive relationship between employees’ perceived abusive supervision and counterproductive work behavior is stronger when leaders have higher levels of organizational political behavior. Furthermore, the moderated mediation model verified whether the path from Machiavellianism to counterproductive work behavior depended on the level of organizational political behavior. Therefore, organizational political behavior can contribute to the impact of leadership’s Machiavellian level on organizational employees’ behavior. This provides inspiration for future research and sustainable development of Chinese SMEs and points out the direction of development. These conclusions are summarized below.

### 5.1 Theoretical implications

The main contribution of this study is to explore and determine how Machiavellianism leads to counterproductive work behaviors. This study not only focuses on the direct impact of Machiavellianism on counterproductive work behavior but also on the key variables that impact Machiavellianism by inducing employees’ counterproductive work behavior.

First, Machiavellianism positively impacts perceived abusive supervision. Thus, the higher the level of Machiavellianism in leadership, the higher perceived abusive supervision. Machiavellianism is characterized by the constant pursuit of profit maximization and a strong desire to control others (Zheng et al., 2017). Moreover, when employees perceive abusive supervision by leaders, they believe that procedures that leaders do not fully develop or protect employees will lead to procedural justice and reduce organizational citizenship behavior (Zellars et al., 2002). When Machiavellian leaders use immoral means, such as deception and control, to pursue personal interests, employees perceive the leaders’ abusive supervision. Therefore, in Chinese SMEs, the higher the level of leaders’ Machiavellianism, the higher the level of perceived abusive supervision.

Second, according to the research results of Uysal et al. (2023), Machiavellianism and counterproductive work behavior have no effect. However, in this study, Machiavellianism positively impacted counterproductive work behavior. Thus, the higher the level of leaders’ Machiavellianism, the more frequent counterproductive work behavior. When leaders make arbitrary decisions that emphasize only their work, employees’ satisfaction decreases (Jin et al., 2022). Moreover, leaders with a high degree of Machiavellianism use employees’ loyalty for their benefit (Amir and Malik, 2016). Machiavellian leaders are better at manipulating employees for their own interests, resulting in employees losing trust in their leaders and increasing counterproductive work behavior (Zhao and Liao, 2013). Therefore, in Chinese SMEs, Machiavellian leaders use immoral means for personal benefit. In this case, employees believe that leadership destroys the normal atmosphere of the organization. It reduces employees’ trust in the organization, promotes counterproductive work behavior, and negatively impacts the organization.

Third, perceived abusive supervision positively affects counterproductive work behavior. This indicates that the higher the level of perceived abusive supervision by their leaders, the higher the level of their counterproductive work behavior. Abusive supervision is a form of superior aggression toward employees and is a typical negative leadership behavior (Jiao and Zhao, 2023). Based on the job demands-resources model, when employees perceive abusive supervision at work, in addition to coping with basic daily work demands, they need to adopt compensatory strategies, that is, exert significant extra physical and psychological energy to cope with abusive supervision, so they struggle to experience the meaning or value in their work (Ma et al., 2023). According to the principles of social exchange theory, leadership behaviors that employees perceive are repaid appropriately, including negative behaviors. Therefore, when employees perceived abusive supervision significantly negatively impacts their psychology and leads to counterproductive work behaviors.

Fourth, perceived abusive supervision mediates the relationship between Machiavellianism and counterproductive work behavior. This suggests that Machiavellianism can directly mediate counterproductive work behavior or affective counterproductive work behavior through perceived abusive supervision. When employees perceive injustice or unethical behavior from an organization, they retaliate (Fu and Zhong, 2020). Thus, Machiavellian leaders are manipulative, like the control employees, and generally have negative views about others, in which case employees perceive abusive supervision by their leaders and lose their enthusiasm for work, leading to burnout and counterproductive work behavior.

Fifth, this study verified the moderating role of organizational political behavior on perceived abusive supervision and counterproductive work behavior. The results indicate that organizational political behavior positively moderates the relationship between perceived abusive supervision and counterproductive work behavior. Thus, the higher the level of interaction between perceived abusive supervision and leaders’ organizational political behavior, the higher the employees’ counterproductive work behavior. When employees encounter abusive supervision, they feel insulted and perceive their organizations as political, which may lead to the loss of their affective resource strands, such as self-esteem and self-confidence; under such circumstances, employees engage in counterproductive work behaviors to protect themselves (Huang et al., 2018). In the case of retaliatory behavior, it is generally believed that an event must trigger a person’s behavior or retaliation (Bowling and Gruys, 2010). Self-interest is central to organizational political behavior, where leaders use political tactics for their own benefit, leading to an increased level of perceived abusive supervision by employees and counterproductive work behaviors. Finally, we demonstrate whether organizational political behavior moderates the mediating effect of perceived abusive supervision. The results indicate that organizational political behavior has a significant moderated mediating role. This implies that the interaction between organizational political behavior and perceived abusive supervision can increase the occurrence of employee counterproductive work behavior. And ultimately, the mediating effect of perceived abusive supervision was moderated on the path between Machiavellianism and counterproductive work behavior. In organizations, leaders who exhibit Machiavellian traits tend to use unethical means to manipulate and control employees for personal gain, which may lead to employees’ perceptions of abusive management by their leaders. In addition, when leaders display high levels of political behavior, this may further reinforce employees’ perception of abusive supervision, thereby increasing the chances of counterproductive work behavior. This provides a basis for exploring or finding more effective methods of inducing counterproductive work behavior in the future.

### 5.2 Practical implication

First, Machiavellian leadership exists in Chinese SMEs. Leaders, as managers and core decision-makers of a firm, can contribute significantly to the sustainable development of the firm (Guo and Su, 2018). Trust is the basis of social exchange (Fu and Zhong, 2020). Therefore, in management practices, leaders should trust their employees fully and reduce deception and control over them to better motivate them to behave in ways that positively impact the organization.

Second, Machiavellianism directly affects unethical pro-organizational behavior and is considered an *a priori* factor for unethical behavior (Kim et al., 2022). Therefore, organizations should focus on developing leaders’ ethical styles through relevant corporate systems and programs. In addition, leaders should establish the right professional values in the organization, help employees work together to achieve organizational goals, protect employees’ interests and resources, increase job satisfaction, reduce organizational labor costs, and reduce the negative effects of organizational brain drain.

Third, when employees perceive abusive supervision by their leaders, they believe that the organization treats them negatively and retaliates through unethical behavior. Employees’ unethical behavior can significantly harm the organization and its sustainable development. The prerequisite for effective control and management of employees’ unethical behavior is to clarify the factors that cause it and the processes that generate it (Zhong et al., 2020). Therefore, organizations should regulate the management styles of leaders to reduce their negative image in employees’ minds. Moreover, standardizing the talent recruitment and selection system, paying attention to the ethical examination of candidates, and strengthening the ethical training of employees to help them recognize and establish correct ethical concepts will positively affect the sustainable development of organizations.

Fourth, Machiavellian leaders achieve their goals by manipulating and deceiving employees, which creates an atmosphere that makes employees feel pressured and develop negative attitudes. The working atmosphere in organizations can determine how employees think and feel about their work environments (Cai and Jin, 2023). Positive working atmospheres increase employees’ willingness to propose solutions to problems, plans for improvement, and constructive ideas and pursue innovation (Jin et al., 2023). Therefore, leaders should create a positive and healthy working atmosphere in the organization so that employees feel sufficient job security to reduce counterproductive work behaviors and improve organizational performance.

Finally, organizational systems and corporate culture determine, to some extent, how organizations approach and deal with political behavior, and organizational political behavior is most likely to occur when resources within the organization are in short supply and existing resource allocation patterns have changed (Qiu, 2015). An excellent corporate culture facilitates the formation of correct work values with employees, increases employees’ trust in their leaders and the organization, and contributes to a fair and just organizational climate. And can increase their sense of autonomy, reduce their concerns about taking risks, and promote their interest in the work itself (Su et al., 2022). Therefore, organizations should establish a set of scientific and standardized rules and regulations and a positive and healthy organizational culture to guarantee the fairness of resource distribution and reduce undesirable political behaviors among people within the organization.

### 5.3 Limitations and future research

Although our study significantly contributes to validating the impact of perceived abusive supervision on the relationship between Machiavellianism and counterproductive work behavior, it has some limitations, as detailed below. We also propose future research directions for organizational sustainability, survival, and innovative behavior.

First, this study examines the role of Machiavellianism in Chinese SMEs. Owing to geographical and cultural differences, it is necessary to conduct similar studies on employees in SMEs in other countries. Because when conducting empirical analyzes on organizational members from different countries and cultures, whether the same results can be obtained is of great research significance (Jin et al., 2023). And compare the findings with those of this study to explore their differences.

Second, this study explored Machiavellianism as a negative variable regarding the harm it poses to organizations. However, Machiavellianism may also have a positive side: individuals high in Machiavellianism can operate more effectively in urgent, unorganized competitive situations, and they usually involve less emotion, which is beneficial for the organization or the individual (Zhao and Liao, 2013). Moreover, Machiavellian leaders are highly adaptable in different situations, analyze problems rationally, and actively take measures to deal with difficulties in fierce competition situations, which can help guide companies to seize opportunities (Deng et al., 2022). Individuals with higher levels of Machiavellianism exhibit a strong desire for control and will do whatever it takes to achieve personal gain, but they strive to control their impulses in pursuit of long-term growth to achieve higher levels of self-management. In addition, Machiavellian leaders tend to skillfully use others or organizational resources to achieve personal goals (Yang et al., 2023). Thus, in organizations where employees are able to deliver high performance or greater benefits, Machiavellian leaders view them as a valuable resource to expand their personal influence and value. In future research, Machiavellianism can be explored as a positive variable to uncover the positive impact it brings to the organization.

Third, we used the 16-item Machiavellianism Scale created by (Dahling et al., 2009). The two items with low values were used in this study. Owing to the differences between Chinese and Western cultures, future research creates Machiavellian scales suitable for Chinese SMEs.

Fourth, this study focused only on the role of perceived abusive supervision as a mediator of the relationship between Machiavellianism and counterproductive work behavior. However, we argue that. in addition to perceived abusive supervision, variables such as employee-leadership match, trust in leaders, and emotional exhaustion mediate this relationship.

Fifth, only organizational political behavior was selected as a moderating variable between perceived abusive supervision and counterproductive work behavior. However, in addition to organizational political behavior, other variables related to individual and organizational aspects should be explored. On the individual side, it is necessary to focus on the political skills of organizational employees, organizational political perceptions, and so on. This is because political skill, which is an interpersonal effectiveness construct, can moderate the process of exchange and feedback interaction between leader and employee (Su et al., 2019), However，organizational political perception can be regarded as an individual’s subjective perception and judgment of organizational political behaviors and phenomena (Li et al., 2020). On the organizational side, future research should explore the organizational political climate. This is because organizational political climate, as employees’ common perception of the degree to which the organization’s internal structure uses the power base to influence decision-making, resource allocation, and goal achievement, will inevitably affect employees’ psychological states and behavioral responses (Zhang et al., 2021). Therefore, it is necessary for us to determine and verify the moderating roles of these variables in future studies.

Sixth, this study focuses on the relationship between Machiavellianism and counterproductive work behavior. Relational conflict may increase employees’ negative perceptions, which are likely to lead to negative attitudes and behaviors (Zhu and Jin, 2023). Future research must explore placing independent variables on the relationship between relationship conflict and counterproductive work behaviors and verify the relationship between them.

Finally, this study was not intentionally categorized, and most participants in the survey were employees. This approach could lead to the over correlation of variables and common method bias(CMB). In future research, it is necessary to strengthen the management of questionnaires to conduct surveys on leadership issues from employees, and leaders should answer questions about employees’ attitudes, behaviors, and performance (Jin et al., 2023), thereby improving the value of data.

## 6 Conclusion

We integrate the results of studies linking Machiavellianism, perceived abusive supervision, organizational political behavior, and counterproductive work behavior based on the relatively few existing studies that have explored the impact of Machiavellianism on employees’ counterproductive work behavior. This study aimed to test this theory. In addition, we extended this to counterproductive work behavior research by measuring both the mediating and moderating effects. We also validated the moderated mediating model to identify the role of counterproductive work behavior in an organization, reduce the harm it causes to the organization, and increase its sustainability and viability. Finally, future research must provide more theoretical perspectives to explain employees’ counterproductive work behaviors in organizations.

## Data availability statement

The raw data supporting the conclusions of this article will be made available by the authors, without undue reservation.

## Ethics statement

Ethical approval was not required for the study involving humans in accordance with the local legislation and institutional requirements. Written informed consent to participate in this study was not required from the participants or the participants’ legal guardians/next of kin in accordance with the national legislation and the institutional requirements.

## Author contributions

HC: Data curation, Methodology, Writing – original draft. LW: Conceptualization, Writing – review & editing. XJ: Conceptualization, Formal analysis, Writing – review & editing.
